# RNF168, a new RING finger, MIU-containing protein that modifies chromatin by ubiquitination of histones H2A and H2AX

**DOI:** 10.1186/1471-2199-10-55

**Published:** 2009-06-05

**Authors:** Sabrina Pinato, Cristina Scandiuzzi, Nadia Arnaudo, Elisabetta Citterio, Giovanni Gaudino, Lorenza Penengo

**Affiliations:** 1 Department of DISCAFF and DFB Center, University of Piemonte Orientale "A. Avogadro", Novara, Italy; 2 Division of Molecular Genetics, The Netherlands Cancer Institute, Amsterdam, The Netherlands

## Abstract

**Background:**

Modulation of chromatin structure has emerged as a critical molecular device to control gene expression. Histones undergo different post-translational modifications that increase chromatin accessibility to a number of regulatory factors. Among them, histone ubiquitination appears relevant in nuclear processes that govern gene silencing, either by inhibiting or activating transcription, and maintain genome stability, acting as scaffold to properly organize the DNA damage response. Thus, it is of paramount importance the identification and the characterization of new ubiquitin ligases that address histones.

**Results:**

We identified and characterized RNF168, a new chromatin-associated RING finger protein. We demonstrated that RNF168 is endowed with ubiquitin ligase activity both *in vitro *and *in vivo*, which targets histones H2A and H2AX, but not H2B, forming K63 polyubiquitin chains. We previously described the presence within RNF168 sequence of two MIU domains, responsible for the binding to ubiquitinated proteins. Here we showed that inactivation of the MIUs impairs ubiquitin binding ability *in vitro *and reduces chromatin association of RNF168 *in vivo*. Moreover, upon formation of DNA double strand breaks induced by chemical and physical agents, RNF168 is recruited to the DNA damage foci, where it co-localizes with γH2AX and 53BP1. The localization of RNF168 at the site of damage highly increases the local concentration of ubiquitinated proteins and determines the prolonged ubiquitination signal.

**Conclusion:**

The RING finger protein RNF168 is a new ubiquitin ligase that functions as chromatin modifier, through histone ubiquitination. We hypothesize a dual function for RNF168. In normal condition RNF168 modifies chromatin structure by modulating ubiquitination of histone H2A. Upon DNA lesions, RNF168 is recruited to DNA damage response foci where it contributes to increase the amount of ubiquitinated proteins, thereby facilitating the downstream signalling cascade.

## Background

Eukaryotic cells have developed efficient ways to modulate the properties of proteins, in order to rapidly respond to variations of external conditions and to face potentially dangerous external events. Among them is the reversible, covalent attachment of modifying groups. Post-translational modifications include small entities such as phosphate or acetyl group, but also entire protein, such as the member of ubiquitin (Ub) family. Ub is a 76 aminoacids polypeptide that has been found appended to many proteins. A cascade of enzymes is required for the ubiquitination reaction. The E1 activating enzyme transfers Ub to an E2 conjugating enzyme that, in cooperation with an E3 Ub ligase, forms a covalent isopeptide bond between the carboxy-terminus of Ub and a lysine residue of the target protein. E3 enzymes are often characterized by the presence of a C3HC4 (RING) finger motif, which binds zinc and is required for Ub ligase activity [1]. Ub contains seven lysine residues that can themselves be substrate of ubiquitination, giving rise to polyUb chains that are differentially decoded by the cell. MonoUb and polyUb conjugates are recognized by proteins through means of short domains called UBDs (Ub binding domains) [2]. The canonical view of ubiquitination as a device to mark proteins for degradation has been evolved to a more multifaceted set of functions, including DNA repair, transcription, cell cycle control, signalling, stress response, viral budding, endocytosis and membrane traffic [3-6].

Since it is one of the most abundant post-translational modifications occurring on histones, ubiquitination functions in the reorganization of chromatin, increasing its accessibility to a number of regulatory factors. Although the role of such modification has been elusive for long, now it is appearing clear that histone ubiquitination serves to regulate gene transcription, either inhibiting or activating it [7-10]. In addition, it has been recently described a critical role for ubiquitination of histones H2A and H2AX in the response to DNA damage. Works from different groups pointed out the relevance of this modification in the correct activation of the signalling cascade triggered by the formation of DNA double strand breaks (DSBs) [11-15]. Activation of ATM induced by DSBs elicits a cascade of phosphorylation and ubiquitination events that promotes the formation of supramolecular complexes, namely the DNA damage response (DDR) foci [16]. DDR foci function in integrating and amplifying the signal, which results in cell-cycle arrest allowing the cell either to repair the damage or to die.

We recently identified and characterized two UBDs present in Rabex-5, a guanine-nucleotide exchange factor for Rab5, named RUZ (Rabex-5 Ub binding zinc finger) and MIU (Motif Interacting with Ub) [17,18]. In particular, MIU is the prototype of a new family of UBDs, since it shows similarity with the well-characterized UIM motif, but it displays a peculiar inverted orientation in the structure [17,18]. Here, we characterized the MIU-containing protein RNF168 as a new E3 Ub-ligase that induces the formation of K63-linked polyUb chains by means of its N-terminal RING finger domain. We demonstrated that histones H2A and H2AX, but not H2B, are substrates of its Ub ligase activity. We found that RNF168 is recruited to DDR foci upon formation of DSBs where it co-localizes with the DDR markers γH2AX and 53BP1, and it promotes sustained ubiquitination at the DDR foci. Finally, we suggest a double mode of recruitment of RNF168 to DDR foci, depending only in part on the functionality of the two MIU domains.

## Results

### RNF168 is a new Ub binding protein that localizes in nuclear structures by means of the MIU domains

**Figure 1:**
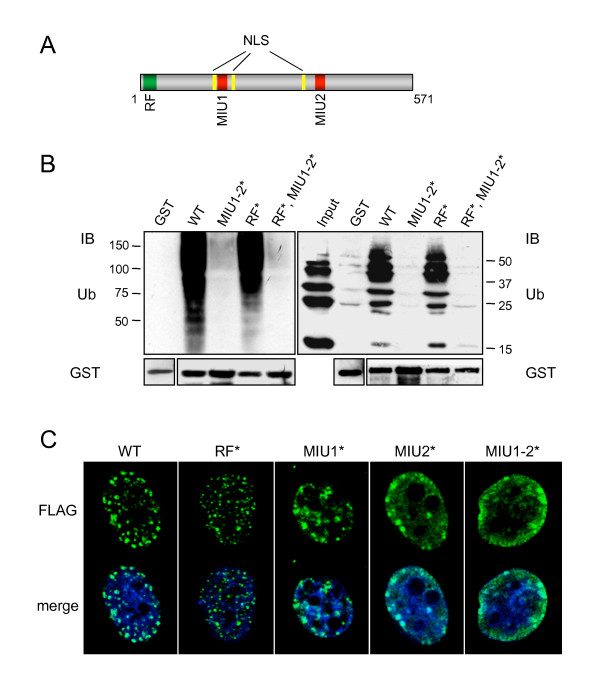
**Ub binding ability and nuclear localization of RNF168**. A) Schematic representation of RNF168: RF (RING finger), MIU (Motif Interacting with Ubiquitin), NLS (Nuclear Localization Sequence). B) *In vitro *pull-down assay was performed using the indicated GST-tagged RNF168 constructs. GST-fusion proteins were incubated with 293T cell lysates (left panel) or synthetic polyUb_2–7 _linked by K48 (right panel) and analysed by SDS-PAGE. Immunoblot (IB) was performed on PVDF membrane treated as described in Methods and immunostained with antibodies directed against Ub and GST. C) 24 hours after transfection with the indicated FLAG-tagged RNF168 constructs, HeLa cells were immunostained with antibodies directed to FLAG. The nucleus was stained with To-Pro 3.

We recently identified and characterized a novel family of UBDs, named MIU [17], which defined a new family of proteins, the MIU-containing proteins. Among them is RNF168 (RING finger protein 168), a 571 amino acid-long protein containing a RING finger motif (RF), two MIU domains and three putative nuclear localization signals (NLS, Figure 1A). We previously demonstrated that the isolated MIU domains of RNF168 were sufficient to interact with Ub [17]. To address the Ub binding ability of the full-length protein, we performed pull-down assays using the GST-RNF168 fusion protein. We found that RNF168 interacts with ubiquitinated proteins derived from cell extracts (Figure 1B left panel), indicating that it is a *bona fide *Ub-binding protein. Inactivation of the two MIU domains, obtained by Ala to Gly substitution at position 179 and 450 of the protein (Ala^179,450^Gly, MIU1-2*), markedly reduced the binding of RNF168 to ubiquitinated proteins. On the contrary, the RF-defective mutant, carrying a double Cys to Ser substitution (Cys^16,19^Ser, RF*) that disrupts the RING finger structure, did not alter Ub binding capability. Then, we performed an Ub-binding assay using synthetic K48-polyUb chains that recapitulated the binding specificity obtained with the cell extracts (Figure 1B right panel). In fact, we observed that RNF168 binding to Ub chains depends on the integrity of the MIUs, while the RF* did not affect the interaction. We can conclude that the MIU domains are required for the binding of RNF168 to ubiquitinated proteins.

To gain insight into the function of the protein, we studied the subcellular localization of RNF168 by immunofluorescence analysis. As expected by the presence of three NLS within its sequence, RNF168 shows nuclear localization (Figure 1C), with a heterogeneous pattern that in most cases appears as marked punctuate staining. Aiming at the identification of the domains responsible for the formation of the RNF168 positive foci, we tested the localization of the mutated forms of the protein defective in the RF and in the MIU domains, carrying either single (Ala^179^Gly, MIU1* and Ala^450^Gly, MIU2*) or double (MIU1-2*) substitutions. As shown in Figure 1C, we found that the integrity of the MIU domains is required for the proper recruitment of RNF168 to the nuclear foci, with a major role played by MIU2. On the other hand, the inactivation of the RF did not exert any effect on the formation of the RNF168 foci, although they appeared smaller then the wild type.

### RNF168 is a Ub ligase with specificity for the formation of K63-linked polyUb chains

**Figure 2:**
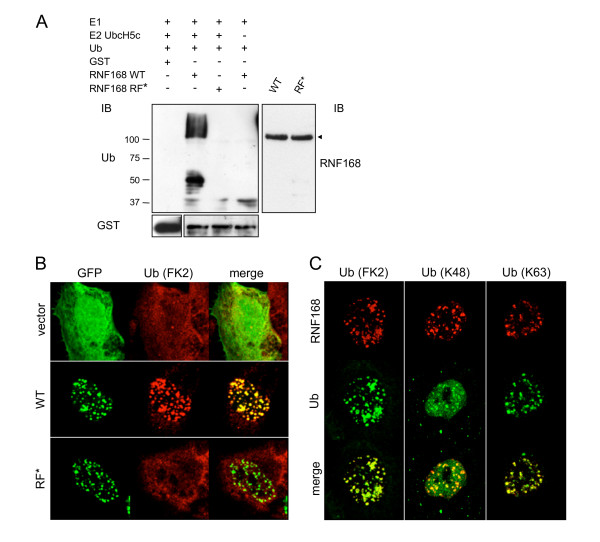
**RNF168 has E3 Ub ligase activity and promotes the formation of K63-linked poly-Ub chains**. A) The intrinsic Ub ligase activity of purified GST-RNF168 was analysed by *in vitro *ubiquitination assay as described in Methods section. Reaction mixtures were resolved by SDS-PAGE and analysed by immunoblotting as shown. The band present at lower molecular weight is likely a degradation product. (◀) indicates the size of GST-RNF168. B) HeLa cells transfected with cDNA encoding the GFP-tagged version of RNF168 and its RF-defective mutant (RF*) were immunostained using antibodies that recognized ubiquitinated proteins (FK2). C) HeLa cells were transfected with FLAG-RNF168 and co-immunostained with antibodies directed to FLAG (red) and to ubiquitinated proteins (green). In particular, we used the antibodies Apu2.07 and Apu3.A8, which specifically recognized K48- or K63-linked polyUb chains, in addition to antibodies FK2.

The RF domain is frequently associated with E3 Ub ligase activity, working in concert with the E2 Ub-conjugating enzyme to ubiquitinate specific substrates [1,19]. Within the E2-E3 complex, the E2 enzyme is endowed with the catalytic activity, while the E3 ligase confers substrate specificity. To investigate the potential intrinsic E3 ligase activity of RNF168, we performed a cell-free *in vitro *ubiquitination assay using either the wild type protein or the RF-defective mutant. We found that RNF168 sustained auto-ubiquitination, and that its E3 ligase activity was dependent on the integrity of the RF domain (Figure 2A).

Then, we asked if RNF168 was able to promote ubiquitination also *in vivo*. We ectopically expressed RNF168 and the RF* mutant in HeLa cells and proceeded with immunofluorescence staining using anti-Ub antibody (FK2) that recognized Ub only when conjugated in chains. Strikingly, almost all cells expressing recombinant RNF168 dramatically increased the amount of ubiquitinated proteins in the nucleus (Figure 2B; [see additional file 1]), which interestingly co-stained with RNF168. As predicted by *in vitro *studies, the RF-defective form of RNF168 did not increase the amount of polyubiquitinated proteins. Altogether, these results strongly proved that RNF168 is an E3 Ub ligase, whose activity depends on the integrity of the RF domain.

Polyubiquitination on K48 is a well-known hallmark for proteasomal degradation, while other types of Ub linkage serve to finely regulate numerous cellular processes, including endocytosis and trafficking, transcription, cell cycle control, DNA damage response and repair [4,5]. Thus, to further characterize the type of ubiquitination promoted by RNF168, we took advantage of two Ub-chain specific antibodies recently described [20], namely Apu2.07 and Apu3.A8, which specifically recognize the K48- or K63-linked Ub chains, respectively. Immunofluorescence analysis revealed that in the presence of RNF168, the polyubiquitinated proteins were recognized mainly by the K63-linkage specific antibodies, and in minor portion by the K48-linkage specific antibodies (Figure 2C). This result indicates that RNF168 promotes the formation of K63-linked polyUb chains, suggesting its potential involvement in the regulation of nuclear events rather then a function in proteasomal degradation.

### RNF168 is a chromatin-binding protein that physically interacts with histone core

**Figure 3:**
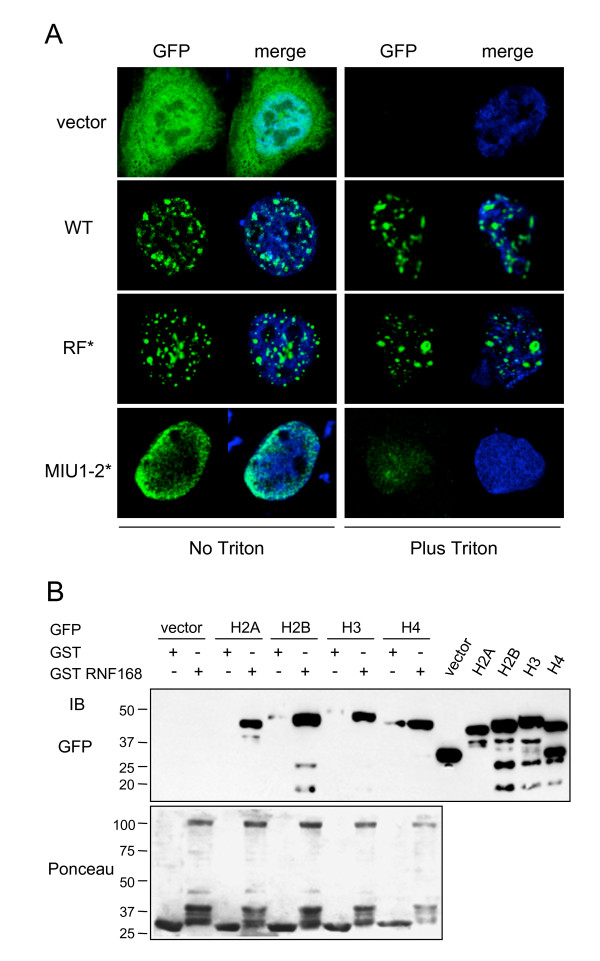
**RNF168 resides in detergent-insoluble structures and interacts with the histone core**. A) Before fixing, HeLa cells transfected with the GFP-tagged indicated constructs or the vector alone, were pre-treated (right panels) or not (left panels) with Triton X-100. The nucleus was stained with To-Pro 3. B) To test RNF168 capability to associate with histones, we performed *in vitro *pull down assay on cell lysate derived from 293T cells expressing the GFP-tagged forms of histones H2A, H2B, H3 and H4. To detect histone binding, the associated proteins were resolved by SDS-PAGE and analysed by anti-GFP immunoblot. To normalize for equal loading, Ponceau-red staining is shown.

To better characterize the RNF168-positive nuclear structures, we treated cells ectopically expressing either GFP-RNF168 or the vector alone with detergent prior to fixation. Interestingly, we found that GFP-RNF168 resides in Triton x-100-insoluble nuclear structures (Figure 3A), suggesting that it is bound to chromatin. Notably, the RF-defective mutant retained its nuclear localization upon detergent extraction, while the mutant MIU1-2* is largely released by the treatment, suggesting that chromatin association of RNF168 is mostly dependent on the integrity of the MIU domains. Comparable results were obtained with the FLAG-tagged version of the protein (data not shown).

This finding prompted us to verify if RNF168 is able to interact with histones. To this purpose, we performed a GST pull-down assay incubating GST or GST-RNF168 with cellular extracts derived from 293T cells expressing a GFP-tagged version of histones H2A, H2B, H3 or H4 (Figure 3B). Interestingly, in all cases we found association with RNF168, suggesting that the interaction likely occurs with the histone core. We can conclude that RNF168 is a chromatin-associated protein that is recruited through the binding with the nucleosome.

### Histones are substrates of RNF168 ligase activity both *in vitro *and *in vivo*

**Figure 4:**
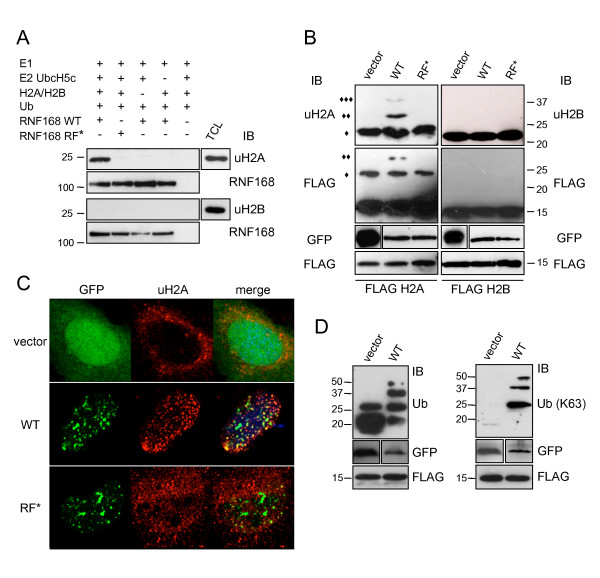
**RNF168 ubiquitinates histone H2A but not H2B, both *in vitro *and *in vivo***. A) The *in vitro *ubiquitination assay was performed with GST-RNF168 wild type and the RF* mutant, using recombinant histones H2A and H2B as substrates. Refer to Methods for details. The reaction mixtures were solved by SDS-PAGE and the immunoblot with antibodies uH2A and uH2B shows the monoubiquitinated forms of histones H2A and H2B, respectively. TCL (Total Cell Lysates) are loaded to validate the signals. Immunoblots directed to RNF168 antibodies were done as control of equal loading. B) 293T cells were co-transfected with GFP alone, GFP-RNF168 WT or RF* together with FLAG-tagged histones H2A (left panels) or H2B (right panels). A small amount (1/20) of the transfected cells were lysed with standard procedure to verify protein expression level (IB using GFP and FLAG antibodies, lower panels). The remaining part was subjected to acid extraction, and the histone component was analysed by SDS-PAGE and immunodecorated as indicated. (◆) indicates the mono-ubiquitinated form of histones H2A and H2B; (◆) di- and (◆◆◆) tri-ubiquitinated forms are visible when the wild type RNF168 is expressed, but not in the presence of the RF* mutant or the vector alone. No signal of di- and tri-ubiquitination was detected for H2B. C) Cells transfected with the indicated GFP constructs were immunostained with anti-uH2A. D) 293T cells were co-transfected with cDNA encoding the GFP-RNF168 (WT) or the vector alone and the FLAG-H2A, and subjected to acid extraction. Proteins were resolved by SDS-PAGE and immunoblotted with FK2 (left panel) and Apu3.A8 (K63-chain specific, right panel) anti-Ub antibodies.

Histone H2A and H2B ubiquitination is emerging as instrumental to finely regulate important nuclear processes [7-10]. Since we have shown that RNF168 is an Ub ligase that interacts with histones, we asked whether it is also able to induce their ubiquitination. Thus, we performed the *in vitro *ubiquitination assay using purified histones H2A and H2B as substrates. To evaluate the ubiquitination status of them we used antibodies that specifically recognize the ubiquitinated form of histones H2A (uH2A) and H2B (uH2B). We found that RNF168 was able to induce ubiquitination of histone H2A in a RF-dependent manner, while it failed to modify histone H2B (Figure 4A).

Next, we aimed at verifying if RNF168 ligase activity targets histones also *in vivo*. To test this, we followed two different experimental approaches. First, we performed biochemical analysis on cells ectopically expressing the wild type RNF168 or the RF defective mutant, together with histones H2A or H2B (Figure 4B). We found that expression of the wild type but not of the RF* mutant markedly increased the level of ubiquitination of H2A (Figure 4B, left panels). Ubiquitination of H2B was not affected (Figure 4B, right panels). Second, we analysed the effect of RNF168 expression on H2A ubiquitination by immunofluorescence analysis (Figure 4C; [see additional file 2]). As a matter of fact, we found that ubiquitination of histones H2A was highly increased in cells where the wild type RNF168 is expressed, while it was not in cells expressing the catalytically inactive RF* mutant (Figure 4C; [see additional file 2]). Furthermore, we aimed at verifying if the RNF168-mediated ubiquitination of histone H2A occurred through K63-linkage, as suggested by immunofluorescence staining (Figure 2C). Acid extracts derived from cells ectopically-expressing GFP and GFP-RNF168, together with FLAG-H2A, showed that RNF168 promoted the formation of polyubiquitinated proteins assembled through K63 (Figure 4D, right panel). Altogether, our data clearly indicate that RNF168 ubiquitinates histone H2A both *in vitro *and *in vivo*, likely inducing K63-linked ubiquitination.

### RNF168 is recruited to DNA damage response foci upon DNA damage

**Figure 5:**
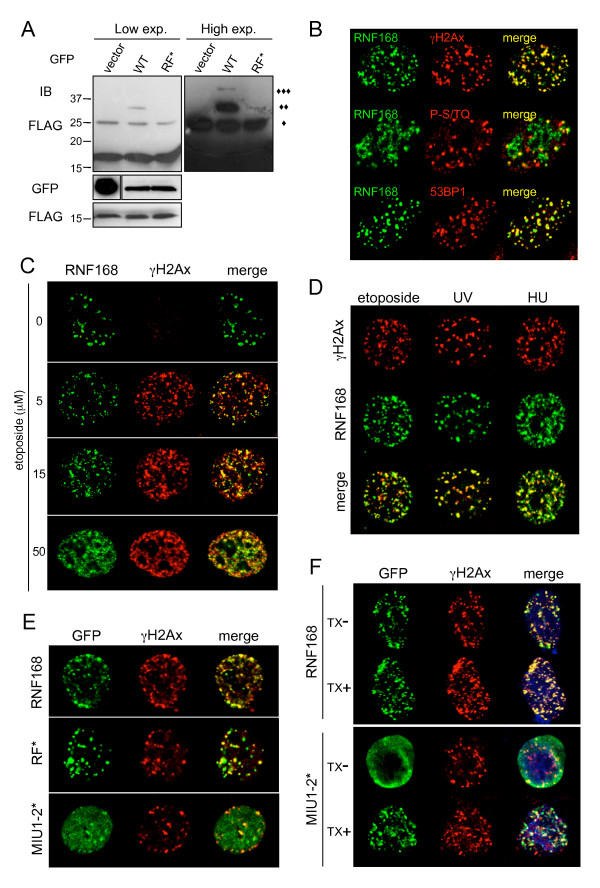
**RNF168 ubiquitinates histone H2AX and is recruited to DDR foci upon DNA damage**. A) *In vivo *ubiquitination of histone H2AX was evaluated in 293T cells co-transfected with GFP alone, GFP-RNF168 WT or RF* together with FLAG-tagged histone H2AX. Lysates were treated as described in Figure 4B. Immunoblot with anti-FLAG antibodies revealed the presence of higher molecular weight proteins compatible with mono- (◆), di- (◆◆) and tri- (◆◆◆) ubiquitinated forms of the histone. Low and high exposure of the immunoblot are shown. B) Cells expressing FLAG-RNF168 were treated with etoposide (5 μM, 1 hour) prior to fixation. To mark DNA damage foci, IF staining was performed using antibodies directed to the phosphorylated form of histone H2AX (γH2AX), to the consensus sequence phosphorylated by the DDR kinases ATM and DNA-PK (P-S/TQ) and the mediator protein 53BP1. C) IF analysis was performed on HeLa cells transfected with FLAG-RNF168 and treated with increasing doses of etoposide, as shown. Immunostaining with anti-γH2AX indicated the number and position of DDR foci. D) DNA damage was induced using different genotoxic agents, such as etoposide (5 μM), UV radiation (UV, 10 J/m^2^) and Hydroxyurea (HU, 2.5 mM). IF analysis was performed as in C. E) HeLa cells transfected with GFP-RNF168, GFP-RF* or GFP-MIU1-2* were treated with etoposide (5 μM) for 1 hr prior to fixation. IF staining performed with antibodies anti-γH2AX revealed the differences in the recruitment at DDR foci of different constructs. F) HeLa cells transfected as in E were incubated with etoposide (5 μM) for 1 hr. To highlight the retention of different constructs in the detergent-insoluble compartments after DNA damage induction, cells were pre-treated (TX+) or not (TX-) with Triton X-100 before fixation. DDR foci are marked by anti-γH2AX immunostaining.

The importance of Ub-mediated network in the DDR and DNA repair is continuously increasing. Ubiquitination of histones H2A and H2AX is crucial to recruit important DDR mediators like 53BP1 and BRCA1 and to promote the signalling cascade elicited by DSBs. Since we have shown that RNF168 is able to ubiquitinate histones, we asked whether RNF168 is also involved in the DDR. First, we tested the ability of RNF168 to induce ubiquitination of H2AX, in addition to H2A. Our biochemical analysis on histones revealed that expression of RNF168 induced ubiquitination of H2AX in a RF-dependent manner (Figure 5A), indicating that RNF168 is an Ub ligase for the histone H2A family. This promising finding highlighted a possible role for RNF168 in the complex series of events that regulate the cellular response to DNA damage. Upon DNA lesions, DDR proteins are rapidly recruited to the damaged site, in order to transduce and amplify the downstream response. Thus, we tested if also RNF168 is recruited to the DDR foci after exposure to genotoxic agents. We found that upon treatment with etoposide, which induces DSBs by inhibition of the Topoisomerase II, RNF168 largely co-localized with markers of the DDR (Figure 5B), as demonstrated by the co-staining with antibodies directed against 53BP1, γH2AX (the phosphorylated form of H2AX), and phospho-S/TQ (the consensus sequence phosphorylated by ATM and DNA-PK). Noteworthy, recruitment of RNF168 to DDR foci upon induction of DSBs is dose-dependent, as demonstrated by treatment of HeLa cells, transfected with FLAG-RNF168, with increasing amount of etoposide (Figure 5C). Furthermore, we examined the recruitment of RNF168 to DDR foci upon treatment with other genotoxic agents, like hydrohyurea and UV light and we found co-localization with γH2AX in all cases (Figure 5D). Then, we investigated the ability of the RNF168 mutants to be recruited to DDR foci, and we found that the RF integrity is not required, since its inactivation did not affect significantly its re-localization (Figure 5E). On the contrary, the MIU-defective mutant displayed a diffused staining, although a small population is concentrated to DDR foci. To better elucidate this point, we thought to extract soluble proteins by detergent treatment (as in Figure 3A) before fixation and proceed with immunofluorescence analysis (Figure 5F). Strikingly, Triton X-100 treatment removed the majority of the MIU1-2* from the nucleoplasm, uncovering the portion of the protein that retained the ability to localize to DDR foci. This finding suggests that it might exist an additional mode of RNF168 recruitment to DDR, which is MIU-independent.

**Figure 6:**
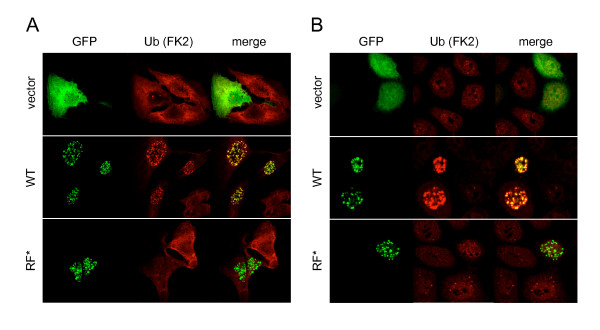
**RNF168 induced prolonged ubiquitination at DDR foci**. A) and B) HeLa cells transfected with GFP-RNF168, GFP-RF* or the vector alone were treated with etoposide (5 μM) for 1 hr and then either fixed (A) or chased for additional 7 hours (B). IF analysis was performed with anti-Ub antibodies (FK2).

In Figure 2C we have shown that RNF168 expression dramatically increased the amount of ubiquitinated proteins in the RNF168 positive foci. Thus, we asked whether we could detect a similar burst of ubiquitination also at the site of DNA damage. As predicted, we found that, upon treatment with etoposide, the ubiquitination signal was highly increased at RNF168 positive foci, but not in the presence of the RF* mutant (Figure 6A).

It has been demonstrated that the DDR can be initiated even in the absence of DNA damage by the sole recruitment of the DDR protein [21,22]. Since we observed that ectopical expression of RNF168 dramatically increased the amount of ubiquitinated proteins in the nucleus, we thought that this event might alone activate the DDR, without genotoxic agents. However, we could not detect any activation of the DDR by simply expressing RNF168 (Figure 5C and data not shown). Nevertheless, we observed a prolonged ubiquitination in cells that expressed RNF168 (Figure 6B) compared to control cells, and to the cell expressing the RF* mutant.

Overall our data strongly indicate that RNF168 is a new histone Ub ligase recruited at the DDR foci upon formation of DSBs, where it considerably increased the amount of polyubiquitinated proteins.

## Discussion

Ubiquitination is a versatile intracellular signalling system largely diffused and with a broad spectrum of possible effects. Originally considered as a universal mark to target proteins for degradation, now its importance is well recognized in a plethora of cellular processes. In fact, ubiquitination is able to regulate ordinary nuclear events, like transcriptional regulation, or to counteract exceptional harmful situations, such as the formation of DNA DSBs induced by genotoxic agents. In both cases, a protagonist role is played by histones, since it has been demonstrated that ubiquitination of histones significantly impacts the regulation of gene silencing and the maintenance of genome stability.

Here, we identified and characterized a new RING finger protein, RNF168, which is endowed with Ub ligase activity, inducing the formation of K63-polyUb chains. RNF168 localizes in chromatin-associated structures, and it promotes ubiquitination of histones belonging to the H2A family. Upon DNA damage induced by chemical or physical agents, RNF168 is recruited to DDR foci, where it significantly increases local concentration of ubiquitinated proteins. In this way, RNF168 might be able to alter the highly ordered structure of chromatin, thereby permitting the access of regulatory factors and creating the docking site for UBD-containing proteins.

The ubiquitination status of histones is known to influence gene transcription, but the exact consequence of that is still not well understood. It has been recently demonstrated that in *Xenopus laevis *ubiquitination/deubiquitination of histone H2A regulates the cell-cycle M-phase progression and activation of the HOX gene expression, thereby driving embryonic development [23]. We found that RNF168 ubiquitinates histone H2A but not H2B, assessing its specificity for the H2A family. We wonder whether RNF168, by acting as a modifier of chromatin structure, is responsible for the activation or the inhibition of specific genes, and, if this is the case, which is the consequence of such regulation.

Another aspect raised from this study is the issue of localization. Activation of DDR pathways does not require broken DNA, but can be elicited by stable association of single repair factors with chromatin. This has been demonstrated for some DDR proteins, like MDC1, NBS1, MRE11 and ATM, which work as sensor or transducer of damage, but not for downstream effectors like CHK1 and CHK2 [21,22]. We reasoned that expression of RNF168, by increasing the ubiquitination of H2A and H2AX on chromatin, might be sufficient to initiate the DDR cascade in the absence of damage. Unlikely, we did not observe any significant activation. Whether this includes the action of other types of post-translational modifications or the recruitment of additional factors is still not known.

During the preparation of this manuscript, two different groups reported the role of RNF168 at the DSBs induced by ionizing radiations [24,25]. They showed that it functions as Ub ligase downstream the Ub ligase RNF8, by amplifying the ubiquitination signal leading to accumulation of 53BP1 and BRCA1 at the site of damage. They demonstrated that recruitment of RNF168 to DDR foci is mediated by the binding of the two MIU domains to uH2A. Although we obtained very similar results, there are some important differences. We found that in the absence of genotoxic treatment, ectopical expression of RNF168 can increase the amount of ubiquitinated proteins and in particular of histone H2A and H2AX. This effect is unlikely due to the over-expression of the protein since it has been observed in cells expressing different amount of recombinant proteins, and because it is strictly dependent on the integrity of the RF domain. Moreover, we recognized a major role played by the two MIUs (mainly the MIU2, data not shown) in the proper recruitment of RNF168 to the DDR foci upon DNA damage, as observed by Stewart et al. and Doil et al. [24,25]. Conversely, we found that a small but significant population of the MIU-defective mutant still localizes to DDR foci. This result might suggest an additional mode of recruitment to DNA lesions, which is MIU-independent. It will be relevant to identify the molecular partner of RNF168 that cooperates in its re-localization to DNA damage structures and might be crucial for the full activation of the DDR program.

Noteworthy, Durocher and colleagues identified two mutations in *RNF168 *gene as responsible of the RIDDLE (radiosensitivity, immunodeficiency, dysmorphic features and learning difficulties) syndrome, a recently described immunodeficiency disorder [25,26]. The mutations identified by the authors give rise to two different truncated proteins, which impede 53BP1 accumulation at DDR foci and hence the proper activation of DDR. Both mutants retain the RF domain, responsible for the Ub ligase activity, but lack the MIU2, indicating that both the Ub binding and the ligase activity are required for the full RNF168 activity. Our finding that the MIU-defective mutant is still partly recruited to the DDR sites suggests that the impairment of the MIU domains might generate a partially functional protein.

Overall, these findings testified the enormous relevance of proteins that change chromatin structure on the onset of human diseases and tumorigenesis. A systematic search for molecular alterations addressing this important class of proteins will help to elucidate the pathogenesis of the human syndromes that are still orphans.

The DSBs are the most harmful lesions that occur on the genome, and are induced by external stimuli, like physical and chemical agents, or can form spontaneously, during normal DNA replication and V(D)J recombination in immune cells. To repair the damage, cells activate several pathways, depending on the cell-cycle phase, which interact each other and cooperate. The homologous recombination (HR) process uses homologous DNA sequences to repair lesions, and generally works in phase G2, while during phase G1/S the cell exploits the NHEJ to ligate broken DSB ends.

Although the RIDDLE syndrome is characterized by a severe immunodeficiency, suggesting an altered development of the immune system, it did not display any V(D)J recombination defects [26]. Nevertheless, it is important to remark that the two truncated versions of RNF168 present in the RIDDLE patient might retain a residual activity that allow the execution of certain cellular processes. Hence, we speculate that RNF168 might have a double role to counteract the formation of the DSBs and to guarantee genome surveillance, both in the process of HR and in NHEJ.

## Conclusion

We can envision a scenario in which RNF168 might regulate chromatin structure, through ubiquitination of histone H2A, and perhaps of other still unknown factors. In addition, upon formation of DSBs, it is rapidly re-localized to the site of lesion, where it participates in the activation of the checkpoint signalling pathways, through activation of either the non-homologous end joining or the homologous recombinantion.

## Methods

### Cell culture and transfection

293T cells were grown in Dulbecco's modified Eagle's medium (SIGMA-Aldrich) supplemented with 10% fetal bovine serum (GIBCO) and 2 mM L-Glutamine (SIGMA). HeLa cells were grown in MEM (GIBCO) supplemented with 10% fetal bovine serum (GIBCO), 2 mM L-Glutamine (SIGMA), 1 mM Sodium Pyruvate (SIGMA) and 1% non-essential amino acid solution (SIGMA). Plasmid transfections were performed using FuGENE reagent (Roche) for immunofluorescence analysis and Calcium Phosphate method for biochemical studies.

### Antibodies and constructs

Antibodies used were: mouse monoclonal anti-FLAG and anti-FLAG affinity gel (M2, Sigma), mouse monoclonal anti RNF168 (Abcam), rabbit Phospho-(Ser/Thr) ATM/ATR Substrate Antibody (Cell Signaling), mouse monoclonal anti-Ub P4D1 (Santa Cruz) and FK2 (Stressgen Bioreagents), mouse monoclonal anti-ubiquityl-Histone H2A (Upstate), mouse monoclonal anti-ubiquityl-Histone H2B (Upstate), mouse monoclonal anti-GFP (Santa Cruz), rabbit polyclonal anti-GST was home made, anti phospho-Histone H2A.X (Ser139; Upstate). The linkage-specific antibodies directed to K48 and K63 (Apu2.07 and Apu3.A8, respectively) were from Genentec. Mouse anti-53BP1 was a gift from Dr T. Halazonetis.

The full length human RNF168 cDNA was purchased from RZPD (clone IRATp970F1053D) and cloned into pGEX 6P2 (GE Healtcare), FLAG-pcDNA3.1 (Invitrogen), and pEGFP-C1 (Clontech). Site-directed mutagenesis was utilized to introduce aminoacid substitutions. The oligonucleotide sequences are: mutant RF* (C^16,19^S) forward: TCCGAGTGCCAGTCCGGGATCTCCATGGAAATCCTC, and reverse: GAGGATTTCCATGGAGATCCCGGACTGGCACTCGGA. To introduce the point mutations in the MIU domains, we used the oligonucleotides previously described in [17]. cDNA of histone H2AX was obtained by retro-transcription of mRNA derived from HeLa cells and it was cloned into FLAG-pcDNA3.1 (Invitrogen), using the following oligonucleotides: forward: AGGGATCCTCGGGCCGCGGCAAGA and reverse: TGCGGCCGCTTAGTACTCCTGGGAGGCCT. cDNAs encoding GFP-tagged histones were: peGFP-H2A was a gift of Dr. P.Y. Perche, pBOS-H2BGFP from BD Pharmingen, pBOS-H3GFP and pBOS-H4GFP were a gift of Dr. H. Kimura. H2A and H2B cDNAs were cloned into FLAG-pcDNA3 (Invitrogen). All the constructs were sequence verified.

### Pull-down experiments

Recombinant GST fusion proteins were expressed in *E. coli *strain BL21 pLys by a 3 hours induction with 1 mM IPTG at 37°C. Bacterial cells were harvested, resuspended in PBS supplemented with Protease Inhibitor Cocktail (SIGMA) and 1 mM PMSF and sonicated. Lysates were incubated with 1% Triton X-100 for 30 min at room temperature (RT) and then centrifuged (14000 rpm for 30 min at 4°C). GST-tagged proteins were purified with Glutathione-Sepharose resin (GE Healtcare) as manufacturer's instructions.

For the pull-down experiment with cellular lysates, 293T cells were prepared by resuspending cells in buffer NETN (50 mM TrisHCl, pH 7.5, 500 mM NaCl, 1 mM EDTA, 1% NP-40, 1 mM PMSF, protease inhibitor cocktail (SIGMA), 20 mM Sodium Pyruvate, 50 mM NaF, 1 mM Na_3_PO_4_, 20 μM NEM, 80 U/ml benzonase), and clarified by centrifugation at 13000 rpm for 30 min at 4°C. 10 μg of GST-fusion proteins immobilized onto GSH beads were incubated with lysates for 2 hours at 4°C. Specifically bound proteins were resolved on SDS-PAGE (10%) and transferred onto PVDF membranes (SIGMA). Membranes were treated with denaturing solution (6 M guanidine hydrochloride, 20 mM Tris-HCl, pH 7.4, 1 mM PMSF, 5 μM β-mercaptoethanol) for 30 min at 4°C. After extensive washes in Tris-buffered saline (TBS) buffer, membranes were blocked in TBS buffer containing BSA (5%) over night and then incubated with anti-Ub P4D1 (Santa Cruz) antibody for 1 hour.

The *in vitro *pull-down assay of polyUb_2–7 _linked by K48 was performed as previously described (Penengo, 2006).

### *In vitro *ubiquitination assay

Reaction mixtures contained 0.1 μg human recombinant E1 Ub-activating enzyme (Boston Biochem), 200 ng of purified UbcH5c (provided by Dr E. Maspero, IFOM, Milan), 5 μg of purified GST-RNF168 WT or RF* protein and 2 μg of Ub (home made) in 25 mM Tris-HCl, pH 7.4, 5 mM MgCl_2_, 100 mM NaCl, 1 μM dithiothreitol, 2 mM ATP. The mixtures were incubated at 30°C for 1.5 hours and the reactions were stopped by boiling in Laemmli buffer. Ubiquitination was detected by anti-Ub (P4D1) immunoblotting. For the *in vitro *ubiquitination using histones as substrates, we also added 2 μg of recombinant H2A/H2B. Expression constructs for *Xenopus laevis *H2A and H2B were kindly provided by Dr. K. Luger and the recombinant proteins were purified and reconstituted as described [27].

### *In vivo *detection of ubiquitinated histones

293T cells were co-transfected with GFP-RNF168 WT or RF* and FLAG-tagged histones. Acid extraction of histones was performed as previously described [28]. Lysates were analysed by SDS-Page on 15% gel of polyacrylamide and subjected to immunoblot analysis as indicated.

### Immunofluorescence analysis

HeLa cells expressing GFP or FLAG-tagged RNF168 constructs were grown on glass coverslips. Cells were fixed in 4% paraformaldehyde. Fixed cells were permeabilized by a 7-min treatment with 0.5% Triton X-100 in BSA, blocked with PBG (PBS, BSA, gelatin) for 1 hour and immunoprobed with the appropriate primary antiboby for 1 hour at RT. Incubation with secondary antibodies (Alexa fluor 488 goat anti-mouse or anti-rabbit IgG, Alexa fluor 546 goat anti-mouse or anti-rabbit IgG, all from Invitrogen) was performed for 30 min at RT. Nuclei were stained with 0.2 μM To-PRO for 10 min. Images were acquired by confocal scanning laser microscope (Leica TCS2; Leica Lasertechnik, Heidelberg, Germany).

## Authors' contributions

LP, CS, SP, EC conceived and designed the experiments. LP, CS performed immunofluorescence analysis. LP, SP, CS, NA performed cloning and biochemical experiments. SP carried out the in vitro ubiquitination assays and pull down experiments. CS, SP wrote the methods and the figure legends. LP wrote the core of the paper. GG contributed to the analysis and interpretation of data. All the authors read and approved the final manuscript.

## Supplementary Material

Additional file 1 **Additional Figure 1 RNF168 Ub-ligase activity**. HeLa cells transfected with cDNA encoding the GFP-tagged version of RNF168 and its RF-defective mutant (RF*) were immunostained using antibodies that recognize ubiquitinated proteins (FK2). Click here for file

Additional file 2 **Additional Figure 2 RNF168 ubiquitinates histone H2A *in vivo***. Cells transfected with the indicated GFP-RNF168, GFP-RF* or the vector alone were immunostained with anti-uH2A. Click here for file
