# Supplementary figures and images for: RNF168, a new RING finger, MIU-containing protein that modifies chromatin by ubiquitination of histones H2A and H2AX
Source: BMC Mol Biol. 2009 Jun 5;10:55. doi: 10.1186/1471-2199-10-55 (PMC2699339; doi:10.1186/1471-2199-10-55)

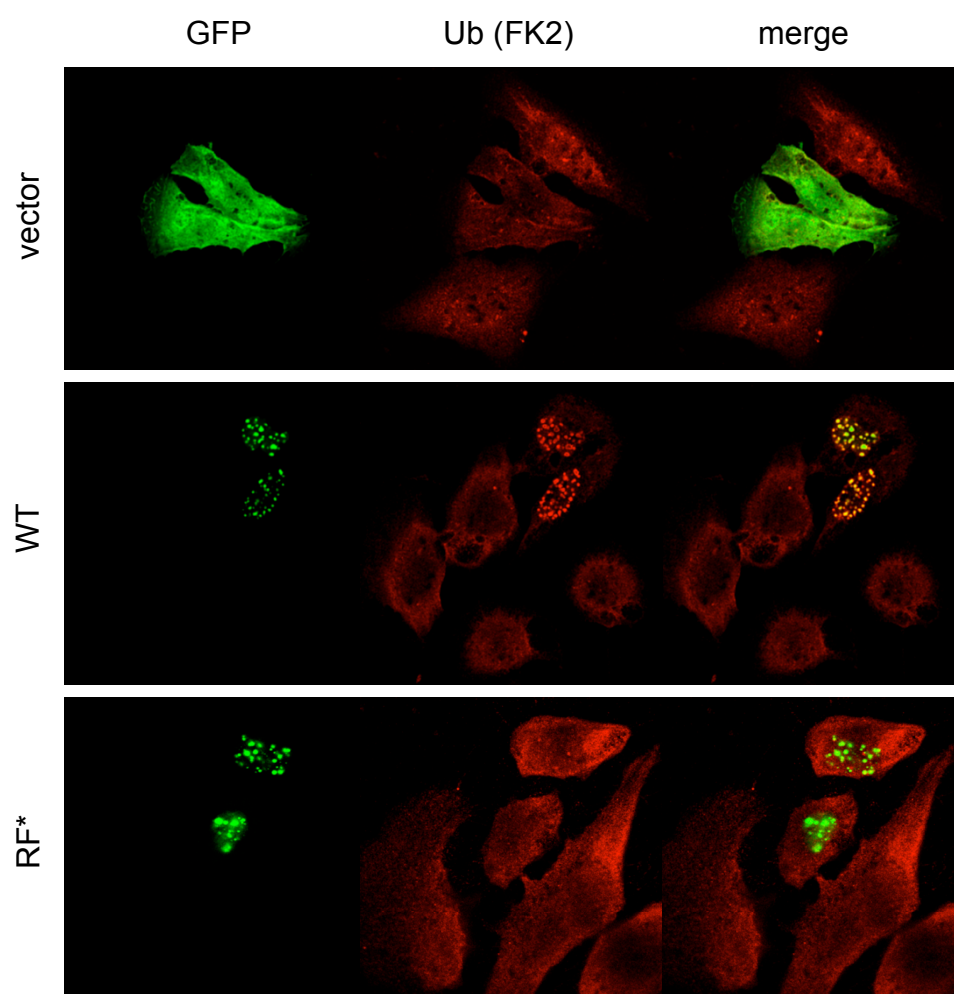

Supplement: Additional file 1 — Additional Figure 1 RNF168 Ub-ligase activity. HeLa cells transfected with cDNA encoding the GFP-tagged version of RNF168 and its RF-defective mutant (RF*) were immunostained using antibodies that recognize ubiquitinated proteins (FK2). [file 1471-2199-10-55-S1.pdf]

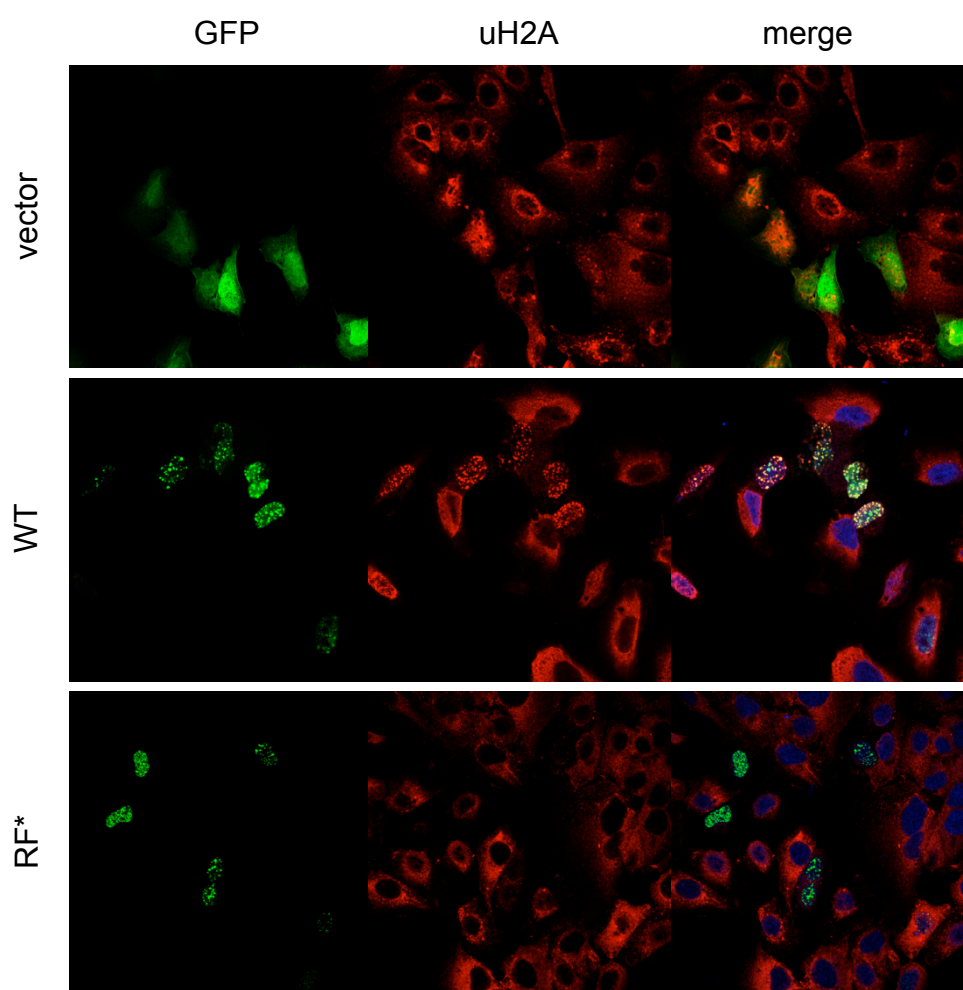

Supplement: Additional file 2 — Additional Figure 2 RNF168 ubiquitinates histone H2A in vivo. Cells transfected with the indicated GFP-RNF168, GFP-RF* or the vector alone were immunostained with anti-uH2A. [file 1471-2199-10-55-S2.pdf]
